# Effect of Various Dynamic Shear Rheometer Testing Methods on the Measured Rheological Properties of Bitumen

**DOI:** 10.3390/ma16072745

**Published:** 2023-03-29

**Authors:** Maya Sheidaei, Anders Gudmarsson, Michael Langfjell

**Affiliations:** 1 Department of Technology and Society, Lund University, P.O. Box 118, 221 00 Lund, Sweden; 2 WSP AB, 121 77 Johanneshov, Sweden; 3 Peab Asfalt AB, 422 46 Hisings-Backa, Sweden

**Keywords:** dynamic shear rheometer (DSR), complex shear modulus, phase angle, sample preparation, rheology, bitumen, bonding temperature, heating temperature, trim

## Abstract

A 2^3^ factorial design experiment was conducted to study the influence of pre-heating temperature (HT) for manufacturing sample, bonding temperature (BT) onto rheometer, and trimming state (Trim) of the sample on complex shear modulus (G*) and phase angle (δ) using a dynamic shear rheometer on unmodified bitumen of types 50/70, 70/100, and two 160/220 from various sources. In addition, the black diagram and 2S2P1D model were used to evaluate the viscoelastic properties of bitumens. Findings show that the G* is more sensitive to the changes than the δ. Additionally, it was found that the 8 mm parallel plate diameter had a higher sensitivity to the trimming state than the 25 mm. The tested factor HT generally did not have a statistically significant impact on the results of the tested materials, except for 160/220_I. At practically all the temperatures tested for 50/70 and 160/220_II, the G* dropped by increasing the factor BT from a lower to a higher value. The Trim:BT interaction has the greatest impact on all materials and temperatures on G*, except for 160/220 at lower temperatures. However, in the case of δ, the Trim:BT interaction has the most significant effects for 70/100 and 160/220_II. The black diagrams show no discernible differences, which may be a result of the limited range of changes made to the variables.

## 1. Introduction

Bitumen is a complex material composed of hydrocarbon molecules with small amounts of heteroatoms, the dominant components of which influence the rheological and thermophysical properties of bitumens [1]. The properties of bitumen vary depending on the crude oil source, the manufacturing method, the addition of additives, and other chemical-physical treatments [2,3]. The quality of bitumen is currently defined in many countries using specifications based on mechanical tests such as needle penetration [4] and ring and ball softening point test [5]. The stress–strain behavior of bitumen at various temperatures and frequencies is not characterized by these tests, despite their ease of understanding. This aspect is considerably more crucial when evaluating modified bitumen since additives alter the frequency and temperature dependence [6,7,8]. The complex shear modulus (G*) is one of the most used rheological properties of bitumen, which is defined as G* = |G*|e*^iδ^*. The dynamic shear modulus |G*| describes the material’s stiffness; phase angle δ describes the extent of viscous and elastic behavior of the material at a given frequency and temperature, and *i* is the imaginary unit (*i*^2^ = −1). The dynamic shear rheometer (DSR) can be used to determine the complex shear modulus, allowing for a comprehensive performance-oriented characterization of bitumen. The method EN 14770:2012 mainly describes the procedure for preparing samples and conditioning [9]. However, numerous aspects associated with laboratory testing setup could potentially alter the final outcomes and lead to discrepancies in results.

Various studies have shown how variables such as the limit of linear viscoelasticity [10], the equipment sensitivity to measure torque [11], and the sample manufacturing method [12] affect the consistency of results. The effects of different plate diameters and the size of the space between the plates have also been investigated [13,14,15,16], while other research has emphasized the importance of applying the same thermal treatment to achieve acceptable precision on the complex modulus, phase angle, and the Strategic Highway Research Program SHRP parameter (G*/sin δ) [17,18,19]. Numerous investigations emphasized the overfilling or underfilling of the gap as well as concerns with reproducibility since the sample diameter is not accurately captured by the DSR in the low-temperature test [20,21].

The aim of this study is to investigate whether and under what conditions the heating temperature for sample manufacturing, the temperature at which the sample bonds onto the DSR, and the radial trimming state of the sample on DSR testing have a substantial impact on the results when the testing setup variables are changed. The interaction effects of the testing conditions will also be explained, as will simple instructions on the test technique and sample and rheometer preparation using parallel plate geometry.

## 2. Materials and Methods

### 2.1. Tested Materials

Table 1 lists the basic properties of the various types of materials employed in this study. The needle penetration test (PEN) according to EN 1426, the softening point (SP) test according to EN 1427, and the bitumen density, which is used to determine the quantity of bitumen required for sample preparation, are provided.

### 2.2. Testing Plan

An Anton Paar MCR302 dynamic shear rheometer with RheoCompass software was used for measuring complex shear modulus and phase angle in an oscillatory-type testing mode using two parallel plate testing geometries. As per the current standard (EN 14770:2012), the linear viscoelastic LVE region is considered the range of strain up to which the values of G′ and G″ differ by less than 5% of the initial value over the chosen shear strain range. To ensure remaining in the LVE range, amplitude sweep (A-sweep) tests were performed at each test temperature which was used in the following Temperature–Frequency-Sweeps (T–f-Sweep) tests. In this study, the strain amplitude limits for the parallel plate with a diameter of 25 mm (PP25) and 8 mm (PP08) were selected at a range of 0.5% (0.005 mm/mm) and 0.1%, respectively.

The Temperature–Frequency-Sweeps (T–f-Sweep) tests were performed for each of the 8 conditions set (runs) and material. Each T-f-Sweep involved 3 samples. For instance, 1 was tested in the temperature range from 30 °C to 0 °C (in descending order) using PP08 and a gap height of 2 mm, and the other 2 were tested in the ranges from 30 °C to 50 °C and 60 °C to 80 °C using PP25 and a 1 mm gap height. The same operator performed 48 T–f-Sweeps per material in total, with 2 repeats, for each tested bitumen over the 8 runs. A frequency range of 0.1 to 10 rad/s was used to collect 10 points from the logarithmic ramp pattern for each constant temperature. The tests were performed at intervals of 10 °C by allowing the material to remain at the test temperature for 15 min within the tolerance of 0.2 °C. Consequently, A-sweep and T–f-Sweep per sample took about 2.5 h.

### 2.3. Design of Experiment

A two-level three-factor factorial designed experiment was conducted to find the effect of the three phases of sample preparation and conditioning such as oven heating temperature for manufacturing sample (HT), the temperature at which the sample bonds onto the equipment (BT), and the radial trim state of manufactured sample on measured data by DSR at temperatures between 0 °C and 80 °C and a frequency of 10 rad/s (1.59 Hz). The recorded data at 80 °C for softer bitumen 160/220 is not considered. The eight combinations of three factors and two levels are shown in Table 2. The sign in parenthesis (− or +) refers to coded values for the factors under study at their low (−) and high (+) levels. The samples were manufactured after heating the material at the planned temperatures (SP + 80 °C and SP + 100 °C) and were bonded onto the DSR at the planned bonding temperature (SP + 0 °C and SP + 25 °C). In without trimming case, the exact amount of sample material is estimated based on the volume of the sample and the density of the materials. To be able to trim the excess material from the sample, the amount of bitumen used in samples with trimming was somewhat higher than in samples without trimming. The upper plate gradually lowered toward the lower plate to reach a bulge around the periphery of the plates at the final gap of 1 mm and 2 mm for PP25 and PP08, respectively. The data values measured for G* and δ are averaged over two genuine replicated runs. Randomization of run order for all 16 runs is performed to ensure that variation between runs is made at the same experimental conditions. The significant parameters were determined manually [22], also with a statistical analysis tool, R (ANOVA test), after estimating the error variance and standard errors of the effects from replicated runs on G* and δ as described in result section.

Furthermore, the rheological test data were modeled using the 2 Spring-2 Parabolic-1 Dashpot (2S2P1D) model [23], and the time–temperature superposition principle (TTSP) [24] to construct Complex modulus and phase angle master curves of the materials tested at an arbitrarily chosen reference temperature of 15 °C. This model consists of seven parameters, and the predicted dynamic shear modulus |G*| can be obtained using the following equation [25]:

(1) $$G_{2S2P1D}^{*}\left(i\omega \tau \right)=G_{0}+\frac{G_{\infty }-G_{0}}{1+\alpha {\left(i\omega \tau \right)}^{-k}+{\left(i\omega \tau \right)}^{-h}+{\left(i\omega \beta \tau \right)}^{-1}}$$

where *i* is a complex number defined by *i*^2^ = −1, *k* and *h* are dimensionless parameters exponents with 0 < *k* < *h* < 1, *α* is a constant parameter related to shape factor, *ω* is angular frequency, G_0_ is the static modulus when *ω*→zero (can be taken as zero since its values are very small and can therefore be neglected), G∞ is the glassy modulus when *ω*→infinity, *β* is a constant parameter related to Newtonian viscosity, and *τ* is characteristic time. In this study, *τ* = *a_T_* (*T_i_*) *τ*_o_ have been estimated by *τ*_o_ which is determined at the reference temperature *τ* (*T_ref_*), and *a_T_* (*T_i_*) a shift factor in the range of temperatures observed in the laboratory *T_i_*, using the William Landel and Ferry (WLF) equation [26]. The well-known method black space diagram is used for visualizing the behavior of tested bitumen, which illustrates complex shear modulus versus phase angle for all tested temperatures.

## 3. Results and Discussion

### 3.1. Evaluation of Main and Interaction Effects

The main and interaction effects of the selected parameters on G* and relevant standard errors were estimated at a frequency of 10 rad/s (1.59 Hz) and a temperature range of 0 °C to 80 °C for the 50/70 and 70/100, and 0 °C to 70 °C for the softer bitumen 160/220_I and 160/220_II. The main effects, two-factor interactions, and three-factor interactions are calculated by averaging individual measures of effects and differences between two averages, as shown below [22]. For convenience, the computation of effects is illustrated just for G*, but the approach for phase angle is similar. The standard test order displayed in Table 2 used to illustrate:

- Trim(G*) = ((G*8 − G*7) + (G*4 − G*3) + (G*6 − G*5) + (G*2 − G*1))/4
- BT (G*) = ((G*3 − G*1) + (G*4 − G*2) + (G*7 − G*5) + (G*8 − G*6))/4
- HT (G*) = ((G*8 − G*4) + (G*7 − G*3) + (G*6 − G*2) + (G*5 − G*1))/4
- Trim:HT (G*) = (G*1 + G*3 + G*6 + G*8)/4 − (G*2 + G*4 + G*5 + G*7)/4
- Trim:BT (G*) = (G*8 + G*5 + G*4 + G*1)/4 − (G*7 + G*6 + G*3 + G*2)/4
- BT:HT(G*) = (G*8 + G*7 + G*2 + G*1)/4 − (G*3 + G*4+ G*5 + G*6)/4
- Trim:BT:HT (G*) = (G*8 + G*5 + G*3 + G*2)/4 − (G*7 + G*6+ G*4 + G*1)/4

The calculation of effects and estimation of standard error are shown only for G* at angular frequency of 10 rad/s and temperature of 60 °C for 50/70. As indicated in Table 3, the average response values are utilized to estimate the variance at each set of conditions. The superscripts represent the randomized run order for all 16 runs displayed in Table 2.

The standard error (SE) of the main or interaction effect is the square root of the average of the estimated variance of 8 observations for an effect, which itself is the difference between the average of 2 levels according to the equation below. The 8 degrees of freedom (DF = 8) in the statistical t-distribution table at the 5% level (α = 0.05) intersect at the significant t-value of 2.3 (Pr(|t_8_| > 2.306) = 0.05). The 95% confidence interval for the estimated effects is given by Effect ± t_8_.SE (effect). Effects that are 2–3 times their standard error are generally sufficiently large to be explained by chance, making them more likely to represent true effects [22], as indicated in bold in Table 4.

(2) $$\mathrm{SE}\;(\mathrm{effect})=\sqrt{\mathrm{Var}\left(\mathrm{effect}\right)}=\sqrt{\left(\frac{1}{8}+\frac{1}{8}\right)\cdot (\sum_{n=1}^{n=8}\frac{diff_{n}^{2}}{2}\;)∕n)}=\sqrt{\left(\frac{1}{4}\right).\left(15937\right)}=63$$

**Figure 1:**
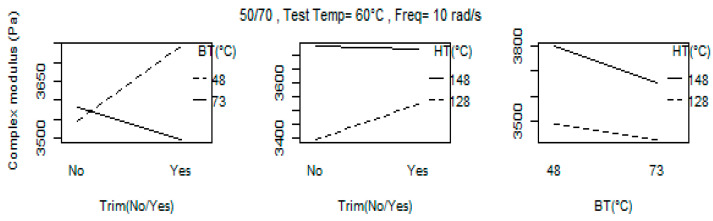
Two-level interaction plots for the G* (*ω* = 10 rad/s) at 60 °C for 50/70.

The two-level interaction is shown in Figure 1. The influence of the main factors cannot be interpreted separately if there is a significant interaction between them, even if the main factors involved are not significant. However, for G* at frequency of 10 rad/s and temperature of 60 °C for 50/70, according to the plots and estimated effects, there is no evidence of interactions, particularly for BT:HT and Trim: HT.

Table 5 shows the F-ratios and the *p*-values from ANOVA. The statistically significant effects of the factors were examined using *p*-values, which is in accordance with results in Table 4. It was found that the main effect HT is statistically significant because the *p*-values from the ANOVA test were less than the confidence limit (α = 0.05) at which the null hypothesis could be rejected.

**Figure 2:**
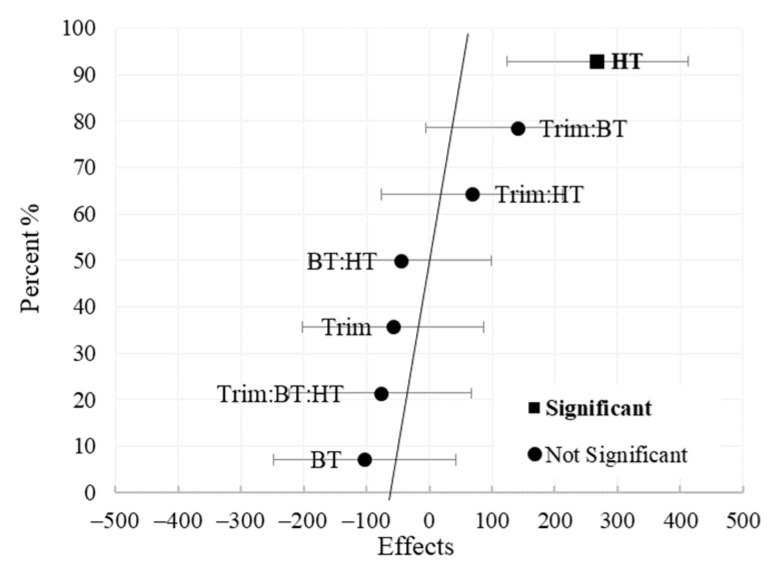
Normal probability of effect estimates at significance level of *α* = 0.05 for the G* (*ω* = 10 rad/s) at 60 °C for 50/70.

Figure 2 displays a normal probability plot of 7 effects obtained from the 2^3^ sample preparation impact experiment on the response G* with a significance threshold of = 0.05. This graphical method aids in determining if the data from the 2^3^ experiment occurred simply as a result of random variation around a mean or if the variations in factor level (−,+) had a real effect on the response. All calculated effects are plotted against a straight line (error line) which represents the normal distribution line. Thus, these 7 effects, which represent 7 contrasts between pairs of averages, would have approximately normal distributions centered at zero and would plot as a straight line on a normal probability scale. On the other hand, it is difficult to attribute random occurrences to the effects that stray from the straight line (outliers) [27]. As shown in Figure 2, the estimated factors BT:HT, Trim, Trim:HT, Trim:BT:HT, and BT fit reasonably well on the straight line. The estimated factor HT evidently deviates the most from the straight line. The estimated factor Trim:BT, however, is the second most distant from the straight line after HT. As a result, it is considered that the main factor HT, denoted by the square, has a stronger impact on the G* at a significance level of 0.05, frequency of 10 rad/s, and temperature of 60 °C for 50/70. Thus, the inferences derived from the normal plot are essentially those made earlier.

The estimated effects and standard errors for all tested materials are shown in Table 6, Table 7, Table 8 and Table 9. The variance of the experimental errors for G* grows with test temperature. To address this problem, the coefficient of variation (CV), which equals the standard deviation (SD) divided by the mean, is used to compare various materials and temperatures. The bonding temperature has the greatest impact on the G* and δ, followed by the oven heating temperature. In the case of 160/220_I, increasing the factor BT from a lower to a higher value increases G* while decreasing δ. However, because the bonding temperatures used are not very high, the higher G* and lower δ cannot be attributed to aging rather than improved adhesion between the bitumen and plates at temperatures slightly over softening point. The variation in oven heating temperature strongly affected the results of the 160/220_I contrary to all other studied materials. Comparing the bonding temperature effect at frequency of 10 rad/s and the first tested temperatures of the 3 samples used for the T–f-Sweep tests (30 °C with PP08, 30 °C with PP25, and 60 °C) indicate that the bonding temperature effect is not significant at the higher test temperature (60 °C) for both G* and δ. Trimming the sample tested at lower temperatures between 0 °C and 30 °C for all tested material tends to significantly increase the δ and decrease the complex modulus, which may indicate higher sensitivity of the smaller parallel plate (PP08) to trimming compared to PP25. Higher G* in the case of without trimming can be due to more amount of excess material left on the periphery of the plates compare to trimmed ones. The BT has an inverse relationship with G* at medium test temperatures for 70/100 and practically all the tested temperatures for 50/70 and 160/220_II. In 2-way interaction, in the case of G*, Trim:BT has the strongest effect on all material and temperatures except for softer bitumen 160/220 at lower temperatures where Trim:HT affects more. This can be due to the low sensitivity of softer bitumen to BT compared to harder bitumen. For δ, the most effective 2-way interaction was Trim:BT for 70/100 and 160/220_II. For 160/220_I, the Trim:HT and for 50/70, the BT:HT are the least important factors for δ. Nevertheless, it may be more relevant to study the interaction effect of Trim:BT at only first tested temperatures after loading the plates with bitumen. Except for 160/220_I, HT does not generally have a statistically significant impact on the results; however, when it interacts with other parameters, it has a substantial impact. This may be attributed to the fact that the range of oven heating temperature (SP + 80 °C and SP + 100 °C) in this study is very small. According to the investigation, the studied parameters have an impact on G* and δ to varying degrees. The factors that have the greatest impact on are 160/220, followed by 50/70 and 70/100.

### 3.2. Master Curves and 2S2P1D Model

Table 10, Table 11, Table 12 and Table 13 illustrate the 2S2P1D parameters that were obtained for each run. As G_0_ for all bitumen is commonly close to zero, the seven parameters can be reduced to six. The average (avg.), standard deviation (SD), coefficient of variation (CV), and coefficient of determination R^2^ (model goodness of fit) are also provided. Statistically significant differences between eight runs and parameters are shown in bold in the tables below.

The sensitivity of each parameter is judged according to the comparison of the correlation of variation (CV) in all runs. The differences of each observation from the mean across the 8 runs are lower for *k*, *h*, and *α* parameters than G_∞_, *τ*, and *β* for all the material except for 160/220_I. The *α* is found to be most inconsistent for the 160/220_I compared to others which could probably be due to the presence of crystalline structure at different temperatures increasing the complexity of the bitumen. It is worth mentioning that *α* can be thought of as an appropriate parameter to assess the occurrence of measurement errors of |G*|. A higher value of *α* indicates that |G*| is exposed to measurement errors and vice versa, which also can be observed from R^2^ values. As the materials become harder, *β* is increased except for 160/220_I which has a significant higher parameter *β*, which means significantly increased viscosity and stiffness modulus at low frequency or high temperature.

The characteristic time *τ* controls the model’s temperature dependence and gives information on how long it takes for a material to relax. A study hypothesized that smaller τ corresponds to softer and less aged binders [15], implying that the higher level chosen for heating temperature (SP + 100 °C) has no effect on the occurrence of aging during sample preparation since all runs and materials with higher heating temperature resulted in smaller τ than runs with lower heating temperature (SP + 80 °C). Similarly, for all investigated materials except 160/220_I, the higher bonding temperature (SP + 25 °C) chosen in this study resulted in a lower τ value compared to the lower bonding temperature (SP + 0 °C).

**Figure 3:**
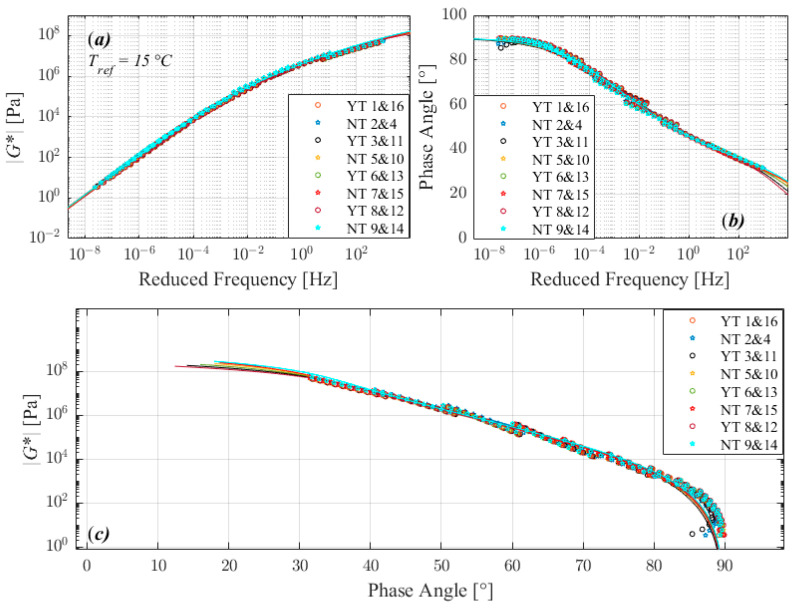
Comparison of master curves of complex shear modulus (**a**), phase angle (**b**), and black diagram (**c**) for 50/70.

**Figure 4:**
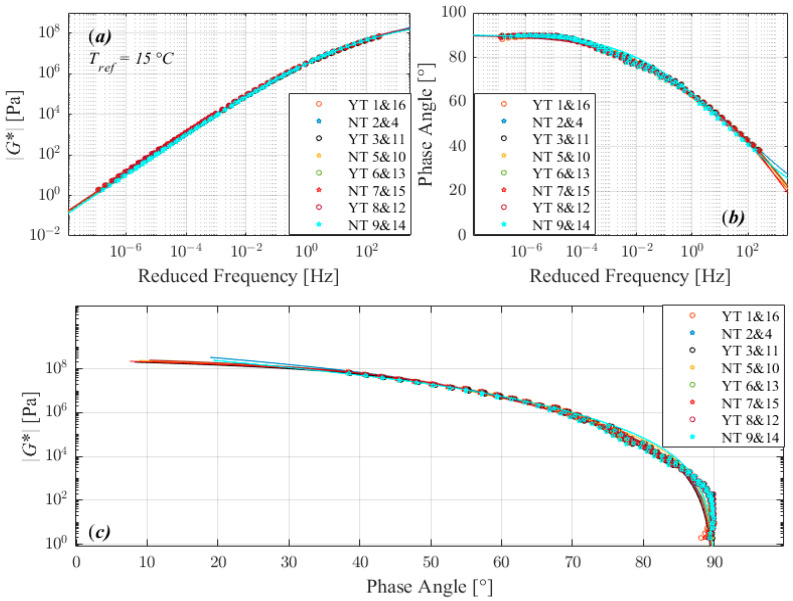
Comparison of master curves of complex shear modulus (**a**), phase angle (**b**), and black diagram (**c**) for 70/100.

**Figure 5:**
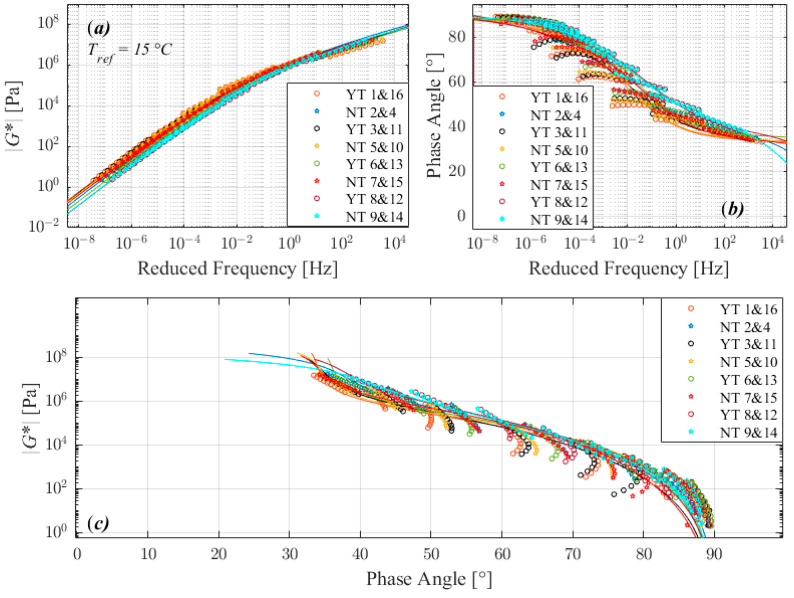
Comparison of master curves of complex shear modulus (**a**), phase angle (**b**), and black diagram (**c**) for 160/220_I.

**Figure 6:**
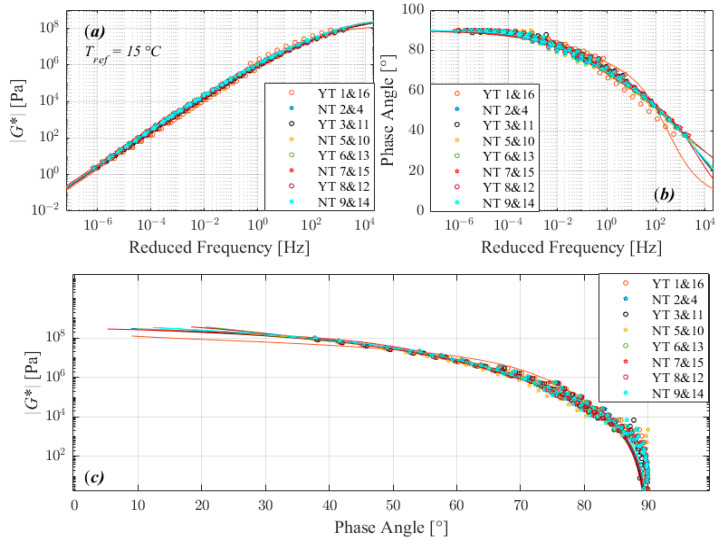
Comparison of master curves of complex shear modulus (**a**), phase angle (**b**), and black diagram (**c**) for 160/220_II.

The trend observed in complex modulus master curves (a), phase angle master curves (b), and black diagrams (c) plots in Figure 3, Figure 4, Figure 5 and Figure 6 suggests that the tests performed with the three different factors with two different level return almost similar measurements. According to the 2S2P1D model, the significant differences observed between different runs are not between 2 extreme set of conditions i.e., 1 and 8, 2 and 7, 4 and 5, or 3 and 6 in standard run order, except for 160/220_I. The 160/220_I showed significant differences between 2 runs of 3 and 6 and significantly higher *β*, *α*, and G_∞_ when the sample was manufactured at a lower temperature, bonded onto DSR at a higher temperature, and trimmed compared to the opposite conditions. The result of the bitumen type 160/220_I (Figure 5) exhibited a different behavior than all other investigated bitumens, similar to the factorial experiment results. The measured complex modulus does not form a continuous master curve in the black diagram (Figure 5c). This type of behavior (discontinuous and tailed complex modulus) has also been seen in previous investigations, which have discussed the possible cause of this divergence [28,29,30,31,32]. Further studies such as fractionation and/or microstructure-based tests are needed to determine whether the divergence can be attributed to the chemical structure of bitumen’s components, as well as their varied dynamics and temperature sensitivity.

## 4. Conclusions

In this study, a 2^3^ experimental design matrix is carried out to evaluate the effect of pre-heating temperature (HT) for manufacturing specimen, bonding temperature (BT) onto rheometer, and trimming state (Trim) on the rheological properties of bitumen when applying dynamic shear rheometer (DSR). Verifications of the observed effects on complex shear modulus (G*) and phase angle (δ) were performed with two replications at various temperatures between 0 °C and 80 °C at frequency of 10 rad/s (1.59 Hz). The tests were executed for 4 neat bitumen of types 50/70, 70/100, and 160/220 from two sources according to EN14770 (2012). The variation in HT strongly affected the results of the 160/220_I contrary to all other studied bitumens. This may be attributed to the fact that the range of oven heating temperature (SP + 80 °C and SP + 100 °C) in this study is very small. Trimming the sample tested at lower temperatures from 0 °C to 30 °C for all tested material tends to significantly increase the δ and decrease the complex modulus, which may indicate higher sensitivity of the smaller parallel plate (PP08) to trimming than the PP25. Increasing the factor BT from a lower to a higher value decreased the G* for practically all the temperatures tested for 50/70 and 160/220_II.

In 2-way interaction, in the case of G*, Trim:BT has the strongest effect on all material and temperatures except for 160/220 at lower temperatures where Trim:HT affects more. For δ, the most effective 2-way interaction was Trim:BT for 70/100 and 160/220_II. The study showed that G* and δ have been affected by the studied factors, the least for 70/100, which is followed by 50/70 and 160/220.

A simple visual analysis of the master curves and black diagram seems to suggest that a different sample preparation method and the corresponding testing procedure used in this study do not necessarily result in a significant effect on the measured rheological properties of the bitumen overall. The 2S2P1D model’s parameters validate the findings, but the large discrepancies across the eight runs do not reveal a consistent pattern among the materials, making it challenging to reach a broad conclusion. Additionally, it appears that the maximum heating temperature and bonding temperature have no aging impacts on the materials. The statistically significant differences were expected to occur between those 2 extreme set of conditions, i.e., in the standard run orders 1 and 8, 2 and 7, 4 and 5, or 3 and 6. However, this significant difference was noticeable only for 160/220_I, between run 3 and 6, when the sample was manufactured at a lower temperature, bonded onto DSR at a higher temperature, and trimmed compared to the opposite conditions. Nevertheless, the results of employing such a narrow range of variation in the sample preparation and conditioning procedures emphasizes the importance of adhering to a more consistent test protocol to improve the repeatability of DSR testing on bitumen.

## Figures and Tables

**Table 1:** Bitumen type and properties.

Sample ID	PEN (0.1 mm) at 25 °C EN 1426	SP (°C) EN 1427	Density at 25 °C kg/m^3^
50/70	61	48.4	1030
70/100	77	46.0	1022
160/220_I	160	41.2	1000
160/220_II	161	39.5	1013

**Table 2:** A two-level three-factor factorial design with two replicates.

Standard Order	Randomized Run Order	Trimming State Trim	Bonding Temp. °C BT	Heating Temp. °C HT
1	6 and 13	Yes (−)	SP (−)	SP + 80 (−)
2	2 and 4	No (+)	SP (−)	SP + 80 (−)
3	1 and 16	Yes (−)	SP + 25 (+)	SP + 80 (−)
4	5 and 10	No (+)	SP + 25 (+)	SP + 80 (−)
5	8 and 12	Yes (−)	SP (−)	SP + 100 (+)
6	9 and 14	No (+)	SP (−)	SP + 100 (+)
7	3 and 11	Yes (−)	SP + 25 (+)	SP + 100 (+)
8	7 and 15	No (+)	SP + 25 (+)	SP + 100 (+)

**Table 3:** Estimating the variance for each set of conditions for G* at frequency of 10 rad/s and temperature of 60 °C for 50/70.

Result from 8 Runs	Average of Duplicate (kPa)	Estimated Variance: (Diff. of Duplicate)^2^/2
G*^6^ and G*^13^	G*1 = (G*^6^ + G*^13^)/2 = 3658.2	(G*^6^ − G*^13^)^2^/2 = (152.7)^2^/2
G*^2^ and G*^4^	G*2 = (G*^2^ + G*^4^)/2 = 3313.1	(G*^2^ − G*^4^)^2^/2 = (419)^2^/2
G*^1^ and G*^16^	G*3 = (G*^1^ + G*^16^)/2 = 3383.4	(G*^1^ − G*^16^)^2^/2 = (99.3)^2^/2
G*^5^ and G*^10^	G*4 = (G*^5^ + G*^10^)/2 = 3475.7	(G*^5^ − G*^10^)^2^/2 = (187)^2^/2
G*^8^ and G*^12^	G*5 = (G*^8^ + G*^12^)/2 = 3825.7	(G*^8^ − G*^12^)^2^/2 = (78.1)^2^/2
G*^9^ and G*^14^	G*6 = (G*^9^ + G*^14^)/2 = 3774.5	(G*^9^ − G*^14^)^2^/2 = (71.9)^2^/2
G*^3^ and G*^11^	G*7 = (G*^3^ + G*^11^)/2 = 3615	(G*^3^ − G*^11^)^2^/2 = (0.4)^2^/2
G*^7^ and G*^15^	G*8 = (G*^7^ + G*^15^)/2 = 3687.3	(G*^7^ − G*^15^)^2^/2 = (3.7)^2^/2
Average of the Estimated Variance of 8 observations:		15,937

**Table 4:** Average Effect with Standard Error for the G* (ω = 10 rad/s) at 60 °C for 50/70.

Factors Effect ± Standard Error	Average
Main Effects	
Trim	−58 ± 63
BT	−103 ± 63
HT	**268 ± 63**
Two-factor interactions	
Trim:BT	140 ± 63
Trim:HT	68 ± 63
BT:HT	−46 ± 63
Three-factor interaction	
Trim:BT:HT	−78 ± 63

**Table 5:** Analysis of variances, ANOVA for the G* (*ω* = 10 rad/s) at 60 °C for 50/70.

Factor	DF	Sum of Square	Mean Square	F Value	*p*-Value (Prob > F)
Trim	1	13,415	13,415	0.84	0.39
BT	1	42,035	42,035	2.64	0.14
HT	1	287,323	287,323	18.03	**0.00**
Trim:BT	1	78,638	78,638	4.93	0.06
Trim:HT	1	18,735	18,735	1.18	0.31
BT:HT	1	8616	8616	0.54	0.48
Trim:BT:HT	1	24,641	24,641	1.55	0.25
Residuals	8	127,495	15,937		
Total	15	600,898			

**Table 6:** Effect estimates and Standard Errors (SE) for 50/70. The significant effects with a 95% confidence interval are shown in bold.

Temp °C	Trim	BT	HT	Trim:HT	Trim:BT	BT:HT	HT:BT:Trim	SE/avg G*
G* (*ω* = 10 rad/s)								
0	**0.13**	**−0.09**	0.03	0.01	−0.04	0.03	0.02	0.02
10	**0.13**	**−0.08**	0.03	0.02	−0.04	0.03	0.02	0.02
20	**0.14**	**−0.07**	0.04	0.03	−0.03	0.02	0.02	0.02
30 PP08	**0.13**	**−0.07**	**0.06**	0.04	−0.03	0.01	−0.01	0.02
30 PP25	−0.03	0.04	0.05	0.04	0.04	0.00	0.00	0.02
40	−0.02	**0.07**	0.05	0.03	0.03	0.00	0.00	0.02
50	−0.01	**0.06**	0.04	0.03	0.02	0.00	0.00	0.02
60	−0.02	−0.03	**0.07**	0.02	0.04	−0.01	−0.02	0.02
70	−0.02	0.02	**0.05**	0.00	0.02	−0.01	−0.03	0.02
80	**−0.02**	0.02	**0.03**	0.00	0.02	−0.01	**−0.03**	0.01
Temp °C	Trim	BT	HT	Trim:HT	Trim:BT	BT:HT	HT:BT:Trim	SE
δ (*ω* = 10 rad/s)								
0	**−0.29**	−0.06	−0.08	0.01	−0.09	−0.07	−0.04	0.11
10	**−0.39**	−0.09	−0.16	−0.10	−0.13	−0.06	−0.02	0.17
20	−0.43	−0.04	−0.28	−0.29	−0.20	0.03	0.14	0.21
30 PP08	−0.35	0.10	−0.38	−0.36	−0.18	0.08	0.30	0.21
30 PP25	0.10	0.23	−0.14	−0.04	−0.31	0.02	0.26	0.15
40	0.00	−0.20	−0.06	0.03	−0.20	−0.02	0.15	0.10
50	−0.01	**−0.26**	−0.03	0.05	−0.07	−0.03	0.05	0.07
60	−0.03	**0.16**	−0.09	0.03	−0.02	0.02	0.01	0.04
70	**0.10**	−0.04	**−0.13**	0.10	−0.01	**−0.09**	0.02	0.04
80	0.04	−0.07	−0.01	0.00	−0.13	−0.03	0.09	0.19

**Table 7:** Effect estimates and Standard Errors (SE) for 70/100. The significant effects with a 95% confidence interval are shown in bold.

Temp °C	Trim	BT	HT	Trim:HT	Trim:BT	BT:HT	HT:BT:Trim	SE/avg G*
G*(*ω* = 10 rad/s)								
0	0.03	0.02	−0.06	−0.03	0.06	0.02	0.02	0.04
10	0.04	0.02	−0.06	−0.03	0.07	0.02	0.02	0.04
20	0.04	0.02	−0.06	−0.03	0.07	0.02	0.02	0.04
30 PP08	0.04	0.01	−0.07	−0.04	0.08	0.03	0.02	0.05
30 PP25	0.00	−0.02	0.03	0.02	0.02	0.00	0.00	0.01
40	0.01	−0.02	0.03	0.02	0.02	0.00	−0.01	0.01
50	0.01	−0.01	0.03	0.02	0.01	0.00	0.00	0.01
60	**0.03**	0.00	0.00	0.01	0.02	0.02	0.00	0.01
70	**0.02**	0.01	0.00	0.01	**0.03**	0.01	−0.01	0.01
80	0.01	0.00	0.00	0.01	**0.02**	0.02	0.00	0.01
Temp °C	Trim	BT	HT	Trim:HT	Trim:BT	BT:HT	HT:BT:Trim	SE
δ (*ω* = 10 rad/s)								
0	**−0.24**	−0.07	0.04	−0.01	**−0.27**	−0.03	0.05	0.10
10	−0.35	−0.06	0.06	−0.03	−0.32	−0.06	0.10	0.15
20	**−0.45**	0.00	0.07	−0.02	−0.28	−0.05	0.15	0.18
30 PP08	−0.43	0.09	0.07	−0.03	−0.16	−0.06	0.17	0.20
30 PP25	−0.19	**0.29**	−0.01	0.00	−0.11	−0.06	0.01	0.09
40	−0.14	0.17	−0.01	0.00	−0.12	−0.05	−0.01	0.08
50	−0.09	0.05	0.00	−0.01	−0.08	−0.03	−0.01	0.06
60	−0.03	−0.01	0.03	−0.01	**−0.07**	**−0.06**	0.02	0.02
70	−0.02	0.02	**0.20**	0.00	−0.02	0.07	0.02	0.07
80	−0.08	−0.26	0.19	−0.12	−0.21	0.11	0.26	0.24

**Table 8:** Effect estimates and Standard Errors (SE) for 160/220_I. The significant effects with a 95% confidence interval are shown in bold.

Temp °C	Trim	BT	HT	Trim:HT	Trim:BT	BT:HT	HT:BT:Trim	SE/avg G*
G*(*ω* = 10 rad/s)								
0	**0.18**	0.07	**−0.12**	−0.05	−0.02	0.05	0.07	0.04
10	**0.17**	**0.19**	**−0.17**	−0.04	−0.03	0.06	0.08	0.04
20	**0.14**	**0.27**	**−0.22**	−0.06	−0.05	0.05	0.06	0.05
30 PP08	0.14	**0.24**	**−0.22**	−0.13	−0.07	0.05	0.02	0.06
30 PP25	0.02	**0.25**	**−0.18**	**−0.14**	**−0.11**	**0.13**	0.05	0.04
40	0.02	**0.13**	**−0.09**	**−0.13**	**−0.09**	**0.14**	0.02	0.02
50	0.01	0.01	0.01	**−0.08**	−0.05	**0.10**	0.00	0.02
60	0.08	−0.10	**0.12**	0.02	−0.04	−0.03	−0.05	0.05
70	0.05	−0.05	**0.10**	0.03	−0.04	−0.03	−0.05	0.04
Temp °C	Trim	BT	HT	Trim:HT	Trim:BT	BT:HT	HT:BT:Trim	SE
δ (*ω* = 10 rad/s)								
0	−0.34	**−2.98**	**1.62**	−0.25	0.33	−0.88	−0.42	0.49
10	0.00	**−4.27**	**2.26**	−0.19	0.54	−1.05	−0.13	0.63
20	0.43	**−4.66**	**2.64**	0.17	0.79	−0.94	0.29	0.75
30 PP08	0.42	**−3.29**	**1.75**	0.67	0.89	−0.34	0.62	0.64
30 PP25	0.11	**−3.18**	**2.42**	**1.49**	**1.89**	**−1.48**	−0.87	0.48
40	−0.06	**−1.28**	**0.75**	**0.97**	**1.13**	**−1.12**	**−0.47**	0.16
50	−0.09	**−0.38**	−0.03	0.25	**0.51**	**−0.54**	**−0.31**	0.12
60	−0.25	0.05	−0.13	−0.08	−0.04	0.04	0.03	0.11
70	−0.29	0.10	−0.27	−0.08	−0.11	0.28	**−0.43**	0.18

**Table 9:** Effect estimates and Standard Errors (SE) for 160/220_II. The significant effects with a 95% confidence interval are shown in bold.

Temp °C	Trim	BT	HT	Trim:HT	Trim:BT	BT:HT	HT:BT:Trim	SE/avg G*
G*(*ω* = 10 rad/s)								
0	**0.15**	**−0.05**	**0.05**	**0.06**	0.02	0.03	0.02	0.01
10	**0.16**	**−0.05**	**0.05**	**0.06**	**0.04**	0.03	0.02	0.01
20	**0.18**	**−0.05**	**0.05**	**0.06**	**0.05**	0.02	0.01	0.02
30 PP08	**0.18**	**−0.05**	**0.05**	**0.06**	**0.05**	0.02	0.00	0.02
30 PP25	0.00	**−0.04**	−0.01	0.03	0.02	0.01	−0.02	0.02
40	0.00	**−0.04**	−0.01	0.03	0.02	0.01	**−0.03**	0.01
50	0.01	**−0.03**	−0.01	**0.02**	0.01	0.01	**−0.03**	0.01
60	0.00	0.00	0.00	0.00	0.02	0.01	−0.01	0.01
70	0.00	0.01	0.00	0.00	**0.02**	0.01	0.00	0.01
Temp °C	Trim	BT	HT	Trim:HT	Trim:BT	BT:HT	HT:BT:Trim	SE
δ (*ω* = 10 rad/s)								
0	**−0.50**	0.02	0.06	0.07	**−0.42**	0.06	0.14	0.09
10	**−0.77**	0.01	0.09	0.12	**−0.52**	0.13	0.26	0.15
20	**−0.88**	0.01	0.09	0.12	**−0.49**	0.19	0.32	0.19
30 PP08	**−0.87**	0.09	0.03	0.03	−0.41	0.18	0.31	0.22
30 PP25	−0.09	**0.33**	0.05	**−0.18**	**−0.11**	−0.05	**0.25**	0.04
40	−0.05	**0.14**	0.01	**−0.10**	**−0.08**	−0.05	**0.18**	0.04
50	−0.03	0.01	−0.01	−0.03	−0.05	−0.02	**0.12**	0.03
60	−0.06	0.03	0.07	0.09	−0.08	−0.08	−0.09	0.07
70	0.02	−0.14	−0.29	−0.01	0.13	−0.38	0.00	0.21

**Table 10:** The 2S2P1D model parameters for all runs for 50/70. Statistically significant differences between runs are denoted in bold.

Run Order	G_∞_	*k*	*h*	*α*	*τ*	*β*	G* R^2^	*δ* R^2^	Log (*τ*)
1 (YT 6&13)	3.5 × 10^8^	0.39	0.68	7.11	6.8 × 10^−4^	83	0.92	1	−3.17
2 (NT 2&4)	**5.9 × 10^8^**	**0.35**	0.67	7.95	**2.8 × 10^−4^**	**141**	0.9	1	**−3.55**
3 (YT 1&16)	**2.4 × 10^8^**	**0.41**	0.7	6.93	**1.8 × 10^−3^**	**47**	0.93	0.99	**−2.75**
4 (NT 5&10)	4.4 × 10^8^	0.37	0.68	**8.06**	5.2 × 10^−4^	92	0.89	0.99	−3.28
5 (YT 8&12)	2.4 × 10^8^	0.4	0.69	**6.74**	1.6 × 10^−3^	51	0.94	1	−2.79
6 (NT 9&14)	5.5 × 10^8^	0.38	0.67	7.95	3.9 × 10^−4^	109	0.82	1	−3.40
7 (YT 3&11)	2.8 × 10^8^	0.4	0.70	7.56	1.4 × 10^−3^	52	0.91	0.99	−2.86
8 (NT 7&15)	5.2 × 10^8^	0.36	0.66	7.72	3.4 × 10^−4^	136	0.90	1	−3.46
avg.	4.0 × 10^8^	0.38	0.68	7.50	8.7 × 10^−4^	88.88	0.90	1.00	−3.06
SD	1.4 × 10^8^	0.02	0.01	0.51	0.00	37.67	0.04	0.01	
CV	0.36	0.06	0.02	0.07	0.70	0.42	0.04	0.01	

**Table 11:** The 2S2P1D model parameters for all runs for 70/100. Statistically significant differences between runs are denoted in bold.

Run Order	G_∞_	*k*	*h*	*α*	*τ*	*β*	G* R^2^	*δ* R^2^	Log (*τ*)
1 (YT 6-13)	3.2 × 10^8^	0.46	0.72	2.83	7.8 × 10^−4^	9	0.99	1	−3.11
2 (NT 2-4)	**8.4 × 10^8^**	0.29	**0.66**	2.92	**6.3 × 10^−5^**	**44**	0.98	1	**−4.20**
3 (YT 1-16)	3.0 × 10^8^	0.35	0.68	**1.54**	3.4 × 10^−4^	20	0.99	1	−3.47
4 (NT 5-10)	2.9 × 10^8^	0.48	0.71	2.98	1.1 × 10^−3^	7	0.99	0.99	−2.96
5 (YT 8-12)	**2.3 × 10^8^**	0.5	0.73	2.81	**1.5 × 10^−3^**	**6**	0.99	0.99	**−2.82**
6 (NT 9-14)	6.7 × 10^8^	0.29	0.67	**3.28**	9.5 × 10^−5^	26	1	0.99	−4.02
7 (YT 3-11)	2.4 × 10^8^	0.49	**0.74**	2.83	1.3 × 10^−3^	7	0.99	0.99	−2.88
8 (NT 7-15)	2.5 × 10^8^	0.5	0.73	2.96	1.4 × 10^−3^	7	0.98	0.99	−2.85
avg.	3.9 × 10^8^	0.42	0.71	2.77	8.3 × 10^−4^	15.75	0.99	0.99	−3.08
SD	2.3 × 10^8^	0.09	0.03	0.52	0.00	13.58	0.01	0.01	
CV	0.59	0.22	0.04	0.19	0.72	0.86	0.01	0.01	

**Table 12:** The 2S2P1D model parameters for all runs for 160/220_I. Statistically significant differences between runs are denoted in bold.

Run Order	G∞	k	h	α	τ	β	G* R2	δ R2	Log (τ)
1 (YT 6-13)	5.1 × 10^9^	0.40	**0.76**	101.10	**1.1 × 10^−5^**	62	0.99	0.96	**−4.97**
2 (NT 2-4)	4.3 × 10^8^	0.41	**0.72**	19.56	2.7 × 10^−4^	43	0.46	0.97	−3.57
3 (YT 1-16)	**5.8 × 10^9^**	0.38	0.74	**128.61**	1.5 × 10^−5^	**115**	0.22	0.95	−4.83
4 (NT 5-10)	1.5 × 10^9^	0.39	0.75	68.25	1.4 × 10^−4^	40	0.38	0.95	−3.86
5 (YT 8-12)	7.2 × 10^8^	0.41	0.73	20.59	4.7 × 10^−5^	62	1	0.97	−4.32
6 (NT 9-14)	**1.8 × 10^8^**	0.44	0.73	**10.63**	**4.2 × 10^−4^**	**31**	0.94	0.99	**−3.38**
7 (YT 3-11)	1.2 × 10^9^	0.39	0.73	44.51	6.5 × 10^−5^	112	0.89	0.95	−4.19
8 (NT 7-15)	1.5 × 10^9^	0.38	0.75	48.17	5.7 × 10^−5^	86	0.88	0.97	−4.24
avg.	2.1 × 10^9^	0.40	0.74	55.18	1.3 × 10^−4^	68.88	0.72	0.96	−3.89
SD	2.1 × 10^9^	0.02	0.01	41.89	0.00	32.32	0.31	0.01	
CV	1.05	0.05	0.02	0.76	1.14	0.47	0.44	0.01	

**Table 13:** The 2S2P1D model parameters for all runs for 160/220_II. Statistically significant differences between runs are denoted in bold.

Run Order	G_∞_	*k*	*h*	*α*	*τ*	*β*	G* R^2^	*δ* R^2^	Log (*τ*)
1 (YT 6-13)	**1.0 × 10^9^**	0.32	0.70	4.19	**1.8 × 10^−5^**	22	1	0.99	**−4.75**
2 (NT 2-4)	3.7 × 10^8^	0.45	0.72	2.24	1.2 × 10^−4^	11	0.99	0.99	−3.93
3 (YT 1-16)	**2.3 × 10^8^**	**0.18**	0.72	**1.58**	1.3 × 10^−4^	11	0.88	0.95	−3.89
4 (NT 5-10)	3.6 × 10^8^	0.45	0.72	2.44	1.1 × 10^−4^	11	1	0.99	−3.94
5 (YT 8-12)	7.8 × 10^8^	0.34	0.7	3.25	2.3 × 10^−5^	23	1	0.99	−4.65
6 (NT 9-14)	5.2 × 10^8^	0.32	**0.67**	1.64	3.5 × 10^−5^	**26**	1	0.99	−4.45
7 (YT 3-11)	3.3 × 10^8^	0.46	0.75	2.87	1.5 × 10^−4^	8	1	0.99	−3.82
8 (NT 7-15)	3.1 × 10^8^	**0.55**	**0.79**	**4.72**	**5.5 × 10^−4^**	**3**	0.95	0.99	**−3.26**
avg.	4.9 × 10^8^	0.38	0.72	2.87	1.4 × 10^−4^	14.38	0.98	0.99	−3.85
SD	2.7 × 10^8^	0.12	0.04	1.14	0.00	8.21	0.04	0.01	
CV	0.56	0.30	0.05	0.40	1.21	0.57	0.04	0.01	

## Data Availability

Data available on request from the corresponding author.
